# The Potential Role of Baseline FT3/FT4 Ratio as a Prognostic Biomarker for Patients With Ischemic Non‐Obstructive Coronary Artery Disease

**DOI:** 10.1002/clc.70331

**Published:** 2026-04-24

**Authors:** Feipeng Wu, Xiandong Zheng, Liju Hong, Qiyan Wu, Rui Yang, Xiaodong Xie, Weijia Ren, Zijian Cai, Yuanyuan Xiao, Youjun Zhou

**Affiliations:** ^1^ Department of Nuclear Medicine, Yan'an Hospital Affiliated to Kunming Medical University, Key Laboratory of Tumor Immunological Prevention and Treatment of Yunnan Province Yan'an Hospital Affiliated to Kunming Medical University Kunming China; ^2^ Key Laboratory of Animal Gene Editing and Animal Cloning in Yunnan Province Yunnan Agricultural University Kunming China; ^3^ Xenotransplantation Engineering Research Center in Yunnan Province Yunnan Agricultural University Kunming China; ^4^ College of Veterinary Medicine Yunnan Agricultural University Kunming China; ^5^ School of Public Health Kunming Medical University Kunming China

**Keywords:** FT3/FT4 ratio, ischemia with non‐obstructive coronary artery disease (INOCA) prognosis, risk stratification, thyroid function

## Abstract

**Background:**

Ischemia with non‐obstructive coronary artery disease (INOCA) is associated with an increased risk of major adverse cardiovascular events (MACE). The relationship between thyroid function and the progression of INOCA remains unclear. This study aims to evaluate the prognostic value of the ratio of serum free triiodothyronine (FT3) to free thyroxine (FT4) in patients with INOCA.

**Methods:**

This study retrospectively included 109 patients who underwent coronary angiography (CAG) and synchronous single‐photon emission computed tomography myocardial perfusion imaging (SPECT MPI) in our hospital due to myocardial ischemia symptoms, and were ultimately diagnosed with INOCA. The enrolled patients were divided into high FT3/FT4 value group and low FT3/FT4 value group based on the median FT3/FT4 ratio (0.317). Regularly follow‐up on MACE in patients, including cardiac death, non‐fatal myocardial infarction, non‐fatal stroke, heart failure, coronary revascularization, and readmission due to angina pectoris.

**Results:**

The incidence of MACE in patients with low FT3/FT4 ratio (< 0.317) was significantly higher than that in patients with high FT3/FT4 ratio (≥ 0.317), and the difference was statistically significant (*p* < 0.05). The proportion of readmission due to unstable angina was significantly increased in the low FT3/FT4 group (*p* < 0.05). Multivariate Cox regression analysis showed that patients with high FT3/FT4 ratios had a lower risk of MACE compared to those with low FT3/FT4 ratios (HR: 0.267, 95% CI: 0.076−0.937, *p* = 0.039).

**Conclusions:**

Low FT3/FT4 ratio was significantly associated with increased MACE incidence in INOCA patients. FT3/FT4 ratio may be a potential biomarker to predict the prognosis of INOCA patients.

## Graphical abstract

A low FT3/FT4 ratio indicates a significant increase in the risk of major adverse cardiovascular events in INOCA patients, or a novel biomarker for prognosis.

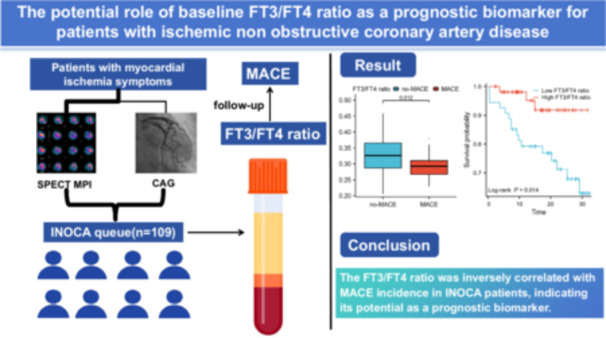

## 1 Introduction

Approximately 70% of patients undergoing coronary angiography (CAG) for angina pectoris do not have obstructive coronary artery disease, and a proportion of these patients are classified as having ischemia with non‐obstructive coronary arteries (INOCA) [1]. The pathophysiological mechanisms of INOCA mainly include, but are not limited to, coronary microvascular dysfunction (CMD) and coronary artery spasm (CAS). Because the evaluation technology of INOCA, whether invasive or noninvasive, has the problems of complex technology, high cost and high risk, the symptoms of patients are often misdiagnosed as non cardiogenic factors, resulting in insufficient diagnosis and treatment [2, 3, 4]. However, multiple studies have demonstrated that INOCA carries an adverse prognosis, as it is associated with an increased risk of patient mortality, myocardial infarction, stroke, poor quality of life, and elevated healthcare costs [5, 6, 7, 8]. These findings further emphasize the important value of standardized clinical management and accurate prognostic stratification of INOCA.

The latest research shows that INOCA is not only limited to the heart, but also one of the manifestations of systemic microvascular dysfunction [9]. Biomarkers, as circulating molecules related to the disease process, can be measured in peripheral blood and can reflect a variety of pathological processes including inflammation, endothelial dysfunction, oxidative stress, coagulation, and so forth, so they may have potential value in the diagnosis, treatment guidance, and prognostic stratification of INOCA [10]. However, effective serum biomarkers for prognostic risk stratification in INOCA patients are still lacking.

The FT3/FT4 ratio serves as a serological marker that more effectively reflects the dynamic metabolic status of thyroid hormones in peripheral tissues compared to other thyroid function indicators such as T3, T4, and TSH. Under pathological conditions like stress or microvascular dysfunction, a decrease in the FT3/FT4 ratio occurs earlier than significant changes in TSH or T4, thereby offering higher sensitivity [11]. Previous studies have shown that FT3/FT4 ratio, as an indicator of thyroid hormone metabolic changes, is closely related to the prognosis of some diseases, such as myocardial infarction with obstructive coronary arteries (MINOCA) and other cardiovascular diseases [12, 13, 14].

However, there is a lack of research on the relationship between FT3/FT4 ratio and INOCA progression. Therefore, this study intends to explore the FT3/FT4 ratio as a potential biomarker to evaluate the outcome risk of INOCA patients, in order to help identify high‐risk groups that need early intervention.

## 2 Methods

### 2.1 Study Population

**Figure 1:**
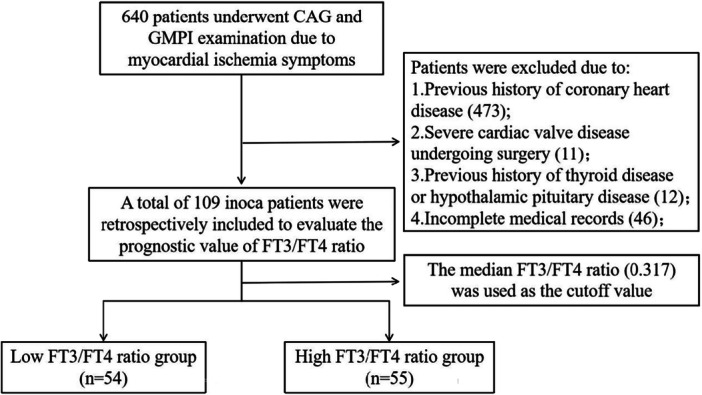
Study flowchart.

This study retrospectively included patients who underwent CAG in our hospital from September 2020 to June 2023 due to myocardial ischemia symptoms and underwent adenosine stress gated nuclear myocardial perfusion imaging during the same period. Inclusion criteria: meet the diagnostic criteria of INOCA [1]; have symptoms and signs of myocardial ischemia; single‐photon emission computed tomography myocardial perfusion imaging (SPECT MPI) suggested myocardial ischemia (objective evidence of myocardial ischemia: reversible radiation defect). Exclusion criteria: (1) Previous clear history of coronary heart disease: percutaneous coronary intervention (PCI)/coronary artery bypass surgery, old myocardial infarction, and so forth; (2) previous history of thyroid disease or hypothalamic pituitary disease; (3) patients with severe inflammation, severe liver dysfunction, and renal insufficiency; (4) patients with severe heart valve disease requiring surgical treatment; (5) patients with malignant tumors with an estimated survival time of less than 1 year; (6) pregnant or lactating women; (7) patients with contraindications to adenosine drug load; and (8) patients with incomplete case data and thyroid function test results. According to the above inclusion and exclusion criteria, 109 eligible patients were finally included (Figure 1).

### 2.2 Data Collection

All baseline data (including demographic characteristics, past medical history, medication information, thyroid function level, etc.) were collected through the electronic medical record system.

### 2.3 Thyroid Function Test Method

All enrolled patients were fasted for 12 h and venous blood samples were collected on an empty stomach in the morning. Blood samples were collected at the time of initial diagnosis, with all samples obtained within 2 weeks before or after CAG and SPECT MPI. The serum was centrifuged and subjected to indoor quality control. The levels of thyroid‐stimulating hormone (TSH), free triiodothyronine (FT3), and free thyroxine (FT4) were measured using an automatic chemiluminescence analyzer (Autolumo A2000, Antu Biological Company, China). The reference range of this laboratory is as follows: TSH: 0.27−4.2 uiu/mL, FT3: 3.5−7.0 pmol/L, FT4: 10−22 pmol/L. Patients with TSH, FT3, and FT4 levels within this reference range were defined as having normal thyroid function.

### 2.4 SPECT MPI Method

This study utilized a 1‐day protocol SPECT MPI, performing adenosine pharmacologic stress imaging first, followed by rest imaging after 4 h. The radiopharmaceutical used was ^99m^Tc‐MIBI. Imaging was performed using a GE Discovery 670pro SPECT/CT scanner. For adenosine stress imaging: adenosine injection was infused via the antecubital vein using a pump for 6 min; ^99m^Tc‐MIBI (295−370 MBq) was intravenously injected at the 3 min mark; a fatty meal was administered 0.5 h later; and image acquisition was performed 1.5 h after the radiotracer injection. For rest imaging: ^99m^Tc‐MIBI (814−925 MBq) was intravenously injected under resting conditions; a fatty meal was administered 0.5 h later, and image acquisition was performed 1.5 h after the radiotracer injection. All images were independently analyzed by two experienced nuclear medicine physicians using a blinded method, one of whom held an advanced professional title in nuclear medicine.

### 2.5 Follow‑Up

The patients were followed up by telephone and medical records. The observed outcome events were major adverse cardiovascular events (MACE), including cardiac death, non‐fatal myocardial infarction, non‐fatal stroke, heart failure, coronary revascularization, and rehospitalization due to angina pectoris. For patients who have readmission records in the medical record system and meet MACE standards, relevant information is directly recorded. For patients without follow‐up hospitalization records, the follow‐up personnel contacted the patients or family members for telephone follow‐up. The follow‐up was conducted every 6 months, and the deadline for follow‐up was June 17, 2024.

### 2.6 Statistical Analysis

Statistical analysis was performed using IBM SPSS 24.0 software. Quantitative data according to normal distribution were expressed as mean ± standard deviation ($\overset{®}{x}$ ± s) and compared between groups by *t*‐test. The quantitative data that did not conform to the normal distribution were represented by the median (M) and the quartile range (Q1, Q3), and were compared by the Mann−Whitney *U* test. Categorical variables were expressed as frequency and percentage (*n*, %), and chi‐square test or Fisher exact test were used for intergroup comparison. Kaplan−Meier survival curve was used to evaluate the prognosis of patients, and log‐rank test was used to compare the differences between groups. Univariate and multivariate Cox regression analysis were used to evaluate the correlation between the FT3/FT4 ratio and composite endpoint events. All tests showed that *p* < 0.05 was statistically significant.

## 3 Results

### 3.1 Baseline Characteristics

According to the median FT3/FT4 ratio, 109 INOCA patients were divided into low FT3/FT4 ratio group (FT3/FT4 < 0.317, *n* = 54) and high FT3/FT4 ratio group (FT3/FT4 ≥ 0.317, *n* = 55). The patients' age ranged from 21 to 78 (55.24 ± 11.59) years, including 61 (56.0%) males. Comparison of the two groups of patients in terms of age, gender, BMI, past medical history (hypertension, hyperlipidemia, diabetes, hyperuricemia), and discharge medications (hypolipidemic drugs, ACEI, ARB, CCB, β‐blocker, nicorandil, trimetazidine, antiplatelet agents, hypoglycemic agents) showed no statistically significant differences (all *p* > 0.05) (Table 1).

**Table 1:** General information of different FT3/FT4 ratio groupings.

Variable	Total (*n* = 109)	Low FT3/FT4 ratio (*n* = 54)	High FT3/FT4 ratio (*n* = 55)	*p* value
Age (years)	55.24 ± 11.59	55.43 ± 12.04	55.05 ± 11.23	0.868
Gender (male/female)	61/48	29/25	32/23	0.638
BMI (kg/m^2^)	24.06 ± 3.33	24.04 ± 3.84	24.08 ± 2.78	0.952
Past history, *n* (%)				
Hypertension	54 (49.5%)	31 (57.4%)	23 (41.8%)	0.151
Hyperlipidemia	31 (28.4%)	17 (31.5%)	14 (25.5%)	0.564
Diabetes	16 (14.7%)	10 (18.5%)	6 (10.9%)	0.297
Hyperuricemia	12 (11.0%)	4 (7.4%)	8 (14.5%)	0.208
Medications, *n* (%)				
Hypolipidemic drugs	74 (67.9%)	33 (61.1%)	41 (74.5%)	0.171
ACEI	15 (13.8%)	8 (14.8%)	7 (12.7%)	0.810
ARB	14 (12.8%)	24 (44.4%)	18 (32.7%)	0.269
CCB	24 (22.0%)	15 (27.8%)	9 (16.4%)	0.182
Beta‐blocker	54 (49.5%)	25 (46.3%)	29 (52.7%)	0.633
Nicorandil	49 (45.0%)	20 (37.0%)	29 (52.7%)	0.069
Trimetazidine	16 (14.7%)	6 (11.1%)	10 (18.2%)	0.262
Antiplatelet agents	49 (45.0%)	23 (42.6%)	26 (47.3%)	0.780
Hypoglycemic agents	16 (14.7%)	9 (16.7%)	7 (12.7%)	0.616

*Note:* Grouping according to the median of FT3/FT4 ratio (low FT3/FT4 ratio: FT3/FT4 < 0.317, high FT3/FT4 ratio: FT3/FT4 ≥ 0.317).

Abbreviations: ACEI, angiotensin converting enzyme inhibitor; ARB, angiotensin Ⅱ receptor blocker; CCB, calcium channel blocker.

### 3.2 Comparison of Prognosis Outcomes in Different FT3/FT4 Ratio Groups

The follow‐up time was 18.4 (9.1, 25.1) months. Nineteen (17.4%) patients had MACE, of which 3 (2.8%) patients received revascularization, 15 (13.8%) patients were readmission due to angina pectoris, and 1 (0.9%) patient had stroke. There were 16 (29.6%) cases and 3 (5.5%) cases of MACE in the low FT3/FT4 ratio group and high FT3/FT4 ratio group, respectively. The difference in the incidence of MACE between the two groups was statistically significant (*p* < 0.05). The proportion of patients readmitted due to unstable angina pectoris in the low FT3/FT4 ratio group was higher than that in the high FT3/FT4 ratio group, and the difference was statistically significant (*p *< 0.05). There was no significant difference in the proportion of revascularization and stroke between the two groups (all *p* > 0.05) (Table 2).

**Table 2:** Comparison of prognosis outcomes in different FT3/FT4 ratio groups.

Variable	Total (*n* = 109)	Low FT3/FT4 ratio (*n* = 54)	High FT3/FT4 ratio (*n* = 55)	*p* value
MACE, *n* (%)	19 (17.4%)	16 (29.6%)	3 (5.5%)	0.001
Revascularization, *n* (%)	3 (2.8%)	3 (5.6%)	0 (0.0%)	0.243
Readmission due to angina pectoris, *n* (%)	15 (13.8%)	12 (22.2%)	3 (5.5%)	0.014
Stroke, *n* (%)	1 (0.9%)	1 (1.9%)	0 (0.0%)	1.000

Abbreviation: MACE, major adverse cardiovascular events.

### 3.3 Univariate Analysis of Different Prognosis in INOCA Patients

Based on whether MACE occurred during follow‐up in INOCA patients, the patients were divided into a MACE group and a control group. Comparisons between the two groups regarding age, gender, BMI, past medical history (including hypertension, hyperlipidemia, hyperuricemia), and discharge medications (including ACEI, ARB, CCD, β‐blockers, nicorandil, trimetazidine, antiplatelet agents, and hypoglycemic drugs) showed no statistically significant differences (all *p* > 0.05). The FT3/FT4 ratio was lower in the MACE group, and the proportion of diabetes was higher in the MACE group, with both differences being statistically significant (*p* < 0.05) (Table 3).

**Table 3:** Univariate analysis of different prognosis in patients with INOCA.

Variable	Total (*n* = 109)	MACE (*n* = 19)	Control (*n* = 90)	*p* value
Age (years)	55.24 ± 11.59	53.42 ± 13.62	55.62 ± 11.16	0.454
Gender (male/female)	61/48	13/7	49/41	0.487
BMI (kg/m^2^)	24.06 ± 3.33	24.51 ± 3.76	23.97 ± 3.25	0.520
Past history, *n* (%)				
Hypertension	54 (49.5%)	11 (57.9%)	43 (47.8%)	0.423
Hyperlipidemia	31 (28.4%)	3 (15.8%)	28 (31.1%)	0.179
Diabetes	16 (14.7%)	6 (31.6%)	10 (11.1%)	0.022
Hyperuricemia	12 (11.0%)	1 (5.3%)	11 12.2%)	0.379
Medications, *n* (%)				
Hypolipidemic drugs	74 (67.9%)	10 (52.6%)	64 (71.1%)	0.117
ACEI	15 (13.8%)	2 (10.5%)	13 (14.4%)	0.652
ARB	14 (12.8%)	9 (47.4%)	33 (36.7%)	0.384
CCB	24 (22.0%)	4 (21.1%)	20 (22.2%)	0.911
β‐blocker	54 (49.5%)	12 (63.2%)	42 (46.7%)	0.191
Nicorandil	49 (45.0%)	8 (42.1%)	41 (45.6%)	0.784
Trimetazidine	16 (14.7%)	4 (21.1%)	12 (13.3%)	0.388
Antiplatelet agents	49 (45.0%)	7 (36.8%)	42 (46.7%)	0.434
Hypoglycemic agents	16 (14.7%)	3 (15.8%)	13 (14.4%)	0.880
Thyroid function				
TSH (uIU/mL)	2.37 (1.54, 3.29)	2.64 (1.55, 3.72)	2.17 (1.49, 3.27)	0.438
FT3 (pmol/L)	5.08 (4.73, 5.32)	4.89 (4.49, 5.20)	5.13 (4.75, 5.37)	0.101
FT4 (pmol/L)	16.15 ± 2.42	17.09 ± 2.18	15.95 ± 2.43	0.061
FT3/FT4 ratio	0.32 (0.28, 0.36)	0.29 (0.27, 0.31)	0.33 (0.29, 0.37)	0.009

Abbreviations: FT3, free triiodothyronine; FT4, free thyroxine; TSH, thyroid‐stimulating hormone.

### 3.4 Cox Regression Analysis and Kaplan−Meier Curve for Different Prognosis of INOCA Patients

**Figure 2:**
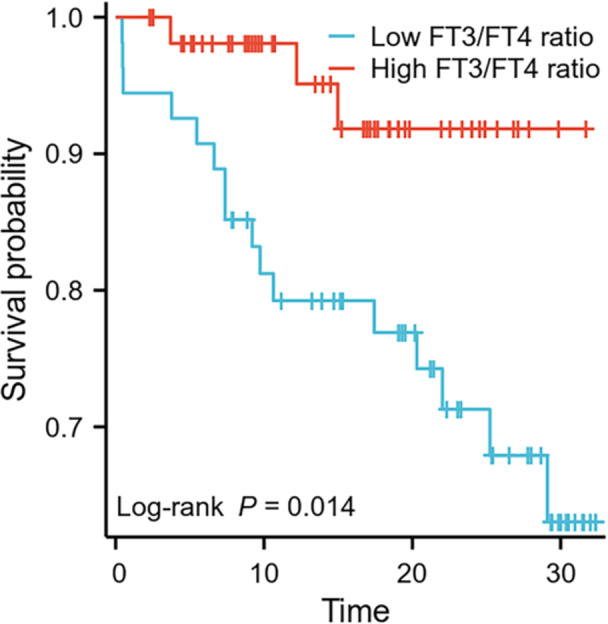
Kaplan−Meier survival curve of different FT3/FT4 ratio groups.

The Kaplan−Meier survival curve showed that the high FT3/FT4 ratio group had a longer mean MACE‐free survival time than the low FT3/FT4 ratio group (mean survival time: 30.0 months [95% CI: 28.2−31.9] vs. 25.5 months [95% CI: 22.5−28.4], *p *= 0.014) (Figure 2).

Univariate Cox regression analysis showed that the FT3/FT4 ratio group (using the low FT3/FT4 ratio group as reference; HR = 0.237, 95% CI: 0.680−0.820, *p *= 0.023) and diabetes (using no diabetes as reference; HR = 2.857, 95% CI: 1.084−7.528, *p* = 0.034) were associated with the occurrence of MACE. Multivariate Cox regression analysis indicated that after adjusting for the influence of diabetes, the FT3/FT4 ratio group (using the low FT3/FT4 ratio group as reference; HR = 0.267, 95% CI: 0.076−0.937, *p* = 0.039) remained associated with the occurrence of MACE (Table 4).

**Table 4:** Multivariate analysis of the different prognosis of INOCA patients.

		Univariate regression analysis			Multivariate regression analysis		
		HR	95% CI	*p* value	HR	95% CI	*p* value
Age (years)		0.978	0.941−1.017	0.269			
Gender	Male	1 (ref)					
	Female	0.840	0.330−2.137	0.714			
BMI (kg/m^2^)		1.026	0.902−1.167	0.698			
Past history, *n* (%)							
Hypertension	No	1 (ref)					
	Yes	1.254	0.502−3.129	0.628			
Hyperlipidemia	No	1 ref)					
	Yes	0.391	0.114−1.346	0.137			
Diabetes	No	1 ref)			1 (ref)		
	Yes	2.857	1.084−7.528	0.034	2.290	0.861−6.091	0.097
Hyperuricemia	No	1 (ref)					
	Yes	0.558	0.074−4.207	0.572			
Medications, *n* (%)							
Hypolipidemic drugs	No	1 (ref)					
	Yes	0.458	0.185−1.129	0.090			
ACEI	No	1 (ref)					
	Yes	0.650	0.150−2.824	0.566			
ARB	No	1 (ref)					
	Yes	1.120	0.451−2.782	0.808			
CCD	No	1 (ref)					
	Yes	0.799	0.264−2.418	0.692			
Beta‐blocker	No	1 (ref)					
	Yes	1.965	0.773−4.998	0.156			
Nicorandil	No	1 (ref)					
	Yes	1.046	0.419−2.609	0.924			
Trimetazidine	No	1 (ref)					
	Yes	2.203	0.726−6.689	0.163			
Antiplatelet agents	No	1 (ref)					
	Yes	0.631	0.248−1.606	0.334			
Hypoglycemic agents	No	1 (ref)					
	Yes	1.049	0.305−3.605	0.939			
Thyroid function							
TSH (uIU/mL)		1.197	0.820−1.747	0.353			
FT3 (pmol/L)		0.844	0.381−1.870	0.676			
FT4 (pmol/L)		1.172	0.980−1.402	0.082			
FT3/FT4 ratio groups	Low	1(ref)			1 (ref)		
	High	0.237	0.68−0.820	0.023	0.267	0.076−0.937	0.039

## 4 Discussion

This study enrolled 109 patients with INOCA. During the 2‐years follow‐up, 19 (17.4%) patients experienced major MACE. Specifically, 3 (2.8%) patients underwent revascularization, 15 (13.8%) patients were rehospitalized due to unstable angina, and 1 (0.9%) patient experienced a stroke. No cases of cardiac death were reported. The incidence of MACE observed in this study was consistent with findings from previous research [15]. Throughout the follow‐up period, the majority of events were related to angina attacks, and no patients experienced acute coronary syndrome (ACS).

FT3 and FT4 are the free forms of triiodothyronine (T3) and thyroxine (T4), respectively, which are physiologically active. Due to their independence from the influence of thyroid‐binding globulin and serum albumin, they offer better sensitivity and stability [16]. T3 is primarily produced by the deiodination of T4 in peripheral tissues. The FT3/FT4 ratio is positively correlated with the activity of deiodinases, reflecting the dynamic metabolic process of T4 conversion to T3 [17, 18]. A decreased FT3/FT4 ratio is associated with adverse outcomes in various cardiovascular diseases. It can serve as a predictor of poor prognosis after PCI in patients with ACS and diabetes mellitus, even when thyroid function is normal [19]. Combining the FT3/FT4 ratio with the GRACE risk score significantly improves the accuracy of prognostic risk assessment for ACS patients after PCI [20]. A low FT3/FT4 ratio also demonstrates high predictive value for cardiovascular mortality and all‐cause mortality in patients with chronic heart failure and is closely related to disease progression [21, 22]. Therefore, when evaluating the prognostic role of thyroid function in cardiovascular diseases, focusing solely on individual thyroid hormone indicators may be insufficient; greater attention should be paid to metrics reflecting thyroid hormone metabolism, such as the FT3/FT4 ratio.

Studies have reported that elevated FT4/FT3 ratio levels are associated with the occurrence of CMD in patients with INOCA [23]. In MINOCA patients with normal thyroid function, low FT3/FT4 ratio levels can predict the risk of MACE in this patient population [13]. However, previous research has primarily focused on the relationship between the FT3/FT4 ratio and coronary microcirculation disorders and MINOCA, with fewer studies investigating the association between the FT3/FT4 ratio and the prognosis of INOCA patients. This study utilized SPECT MPI combined with CAG to diagnose INOCA, which is more accurate than previous methods that assessed myocardial ischemia using electrocardiograms or echocardiograms. The results confirmed that: (1) compared to the high FT3/FT4 ratio group, the incidence of MACE was significantly higher in INOCA patients in the low FT3/FT4 ratio group, primarily driven by an increased occurrence of worsening unstable angina; (2) after adjusting for the influence of diabetes, the FT3/FT4 ratio remained an independent predictor of adverse prognosis in INOCA patients.

Thyroid hormone receptors are widely distributed in myocardial and vascular tissues. Even subtle fluctuations in thyroid hormone concentrations can significantly regulate various physiological functions of the cardiovascular system [24]. The pathophysiological mechanisms underlying the association between the FT3/FT4 ratio and adverse prognosis in INOCA patients remain unclear. Potential mechanisms may include [25, 26, 27]: (1) When the conversion and generation of FT3 are insufficient, its role as an antioxidant factor is weakened, leading to more significant damage to the endoplasmic reticulum under oxidative stress; additionally, reduced FT3 levels hinder myocardial cells' ability to utilize adenosine triphosphate as an energy source, resulting in diminished contractile function; (2) myocardial cells possess T3 receptors that directly regulate cardiac function; a decline in FT3 levels affects myocardial contractility, increases arrhythmia risk, and may exacerbate conditions in heart failure patients, thereby elevating mortality risk; (3) reduced FT3 levels are associated with impaired lipid metabolism, which can elevate the risk of atherosclerosis.

Diabetic patients are at a higher risk of developing endothelial dysfunction, inflammation, and oxidative stress, all of which can lead to impaired microcirculatory function, reduce myocardial blood supply, and further exacerbate the progression of INOCA [28]. In this study, the proportion of diabetes was statistically significant in the univariate analysis but not in the multivariate analysis, which may be related to the relatively small sample size. TSH is the most sensitive indicator reflecting thyroid function. In both univariate and multivariate Cox regression analyses in this study, TSH showed no correlation with the MACE composite endpoint, suggesting that in INOCA patients with normal thyroid function, disease progression may primarily be associated with impaired conversion of T4 to T3.

### 4.1 Limitation

First, patient follow‐up was conducted via telephone, which may introduce recall bias. Second, this study only included the baseline FT3/FT4 ratio without further monitoring changes in thyroid function during the follow‐up period, and the relationship between dynamic changes in the FT3/FT4 ratio and prognosis requires further in‐depth investigation. Third, as a single‐center study with a small sample size, the conclusions are limited, and the effectiveness of the FT3/FT4 ratio in predicting the prognosis of INOCA patients still needs further research confirmation.

## 5 Conclusion

In summary, in INOCA patients with normal thyroid function, the FT3/FT4 ratio may serve as a potential indicator for predicting MACE. Therefore, regular testing and evaluation of the FT3/FT4 ratio could provide a simple and efficient clinical method for assessing and stratifying the prognostic risk in this patient population.

## Author Contributions

F.W. was responsible for experimental design and manuscript writing. X.Z. was responsible for experimental execution and statistical analysis. Q.W., L.H., L.Y., L.Y., and D.C. were responsible for experimental execution. Y.Z. and Y.X. was responsible for experimental design, manuscript review, and funding support.

## Ethics Statement

This study was conducted in accordance with the Helsinki Declaration. Informed consent was obtained from all human participants, and the study protocol was approved by the Ethics Committee of Yan'an Hospital Affiliated to Kunming Medical University (Approval No. 2024‐194‐01).

## Conflicts of Interest

The authors declare no conflicts of interest.

## Data Availability

The data sets used in this study are available from the corresponding author, Professor Zhou Youjun, upon reasonable request (Email: zhouyoujun2023@163.com).

## References

[1] V. Kunadian , A. Chieffo , P. G. Camici , et al., “An EAPCI Expert Consensus Document on Ischaemia With Non‐Obstructive Coronary Arteries in Collaboration With European Society of Cardiology Working Group on Coronary Pathophysiology & Microcirculation Endorsed by Coronary Vasomotor Disorders International Study Group,” EuroIntervention: Journal of EuroPCR in Collaboration With the Working Group on Interventional Cardiology of the European Society of Cardiology 16 (2021): 1049–1069. 32624456 10.4244/EIJY20M07_01 PMC9707543

[2] T. Padro , O. Manfrini , R. Bugiardini , et al., “ESC Working Group on Coronary Pathophysiology and Microcirculation Position Paper on ‘Coronary Microvascular Dysfunction in Cardiovascular Disease’,” Cardiovascular Research 116 (2020): 741–755. 32034397 10.1093/cvr/cvaa003 PMC7825482

[3] C. Vrints , F. Andreotti , K. C. Koskinas , et al., “2024 ESC Guidelines for the Management of Chronic Coronary Syndromes,” European Heart Journal 45 (2024): 3415–3537. 39210710 10.1093/eurheartj/ehae177

[4] M. G. Del Buono , R. A. Montone , M. Camilli , et al., “Coronary Microvascular Dysfunction Across the Spectrum of Cardiovascular Diseases,” Journal of the American College of Cardiology 78 (2021): 1352–1371. 34556322 10.1016/j.jacc.2021.07.042 PMC8528638

[5] M. A. Gdowski , V. L. Murthy , M. Doering , A. G. Monroy‐Gonzalez , R. Slart , and D. L. Brown , “Association of Isolated Coronary Microvascular Dysfunction With Mortality and Major Adverse Cardiac Events: A Systematic Review and Meta‐Analysis of Aggregate Data,” Journal of the American Heart Association 9 (2020): e14954. 10.1161/JAHA.119.014954 PMC7428565 32345133

[6] T. S. Kenkre , P. Malhotra , B. D. Johnson , et al., “Ten‐Year Mortality in the WISE Study (Women's Ischemia Syndrome Evaluation),” Circulation: Cardiovascular Quality and Outcomes 10 (2017): e003863. 29217675 10.1161/CIRCOUTCOMES.116.003863 PMC5728666

[7] H. R. Reynolds , M. H. Picard , J. A. Spertus , et al., “Natural History of Patients With Ischemia and No Obstructive Coronary Artery Disease: The CIAO‐ISCHEMIA Study,” Circulation 144 (2021): 1008–1023. 34058845 10.1161/CIRCULATIONAHA.120.046791 PMC8478858

[8] L. Jespersen , A. Hvelplund , S. Z. Abildstrom , et al., “Stable Angina Pectoris With No Obstructive Coronary Artery Disease Is Associated With Increased Risks of Major Adverse Cardiovascular Events,” European Heart Journal 33 (2012): 734–744. 21911339 10.1093/eurheartj/ehr331

[9] S. Ohura‐Kajitani , T. Shiroto , S. Godo , et al., “Marked Impairment of Endothelium‐Dependent Digital Vasodilatations in Patients With Microvascular Angina: Evidence for Systemic Small Artery Disease,” Arteriosclerosis, Thrombosis, and Vascular Biology 40 (2020): 1400–1412. 32237907 10.1161/ATVBAHA.119.313704

[10] T. Chakrala , R. Prakash , C. Valdes , C. J. Pepine , and E. C. Keeley , “Circulating Biomarkers in Coronary Microvascular Dysfunction,” Journal of the American Heart Association 12 (2023): e29341. 10.1161/JAHA.122.029341 PMC10356037 37301749

[11] Y. Zhou , Q. He , H. Ai , et al., “The Long‐Term Prognostic Implications of Free Triiodothyronine to Free Thyroxine Ratio in Patients With Obstructive Sleep Apnea and Acute Coronary Syndrome,” Frontiers in Endocrinology 15 (2024): 1451645. 39351531 10.3389/fendo.2024.1451645 PMC11439673

[12] X. Lang , Y. Li , D. Zhang , Y. Zhang , N. Wu , and Y. Zhang , “FT3/FT4 Ratio Is Correlated With All‐Cause Mortality, Cardiovascular Mortality, and Cardiovascular Disease Risk: NHANES 2007–2012,” Frontiers in Endocrinology (Lausanne) 13 (2022): 964822. 10.3389/fendo.2022.964822 PMC9433660 36060933

[13] S. Gao , W. Ma , S. Huang , X. Lin , and M. Yu , “Predictive Value of Free Triiodothyronine to Free Thyroxine Ratio in Euthyroid Patients With Myocardial Infarction With Nonobstructive Coronary Arteries,” Frontiers in Endocrinology 12 (2021): 708216. 34394005 10.3389/fendo.2021.708216 PMC8356082

[14] D. Yuan , C. Zhang , S. Jia , et al., “Predictive Value of Free Triiodothyronine (FT3) to Free Thyroxine (FT4) Ratio in Long‐Term Outcomes of Euthyroid Patients With Three‐Vessel Coronary Artery Disease,” Nutrition, Metabolism, and Cardiovascular Diseases 31 (2021): 579–586. 10.1016/j.numecd.2020.10.011 33250369

[15] E. Lampas , K. Syrmali , G. Nikitas , E. C. Papadakis , and S. P. Patsilinakos , “Five‐Year Morbidity and Mortality of Patients With Ischemia With Non‐Obstructive Coronary Artery Disease and Myocardial Single‐Photon Emission Computed Tomography Perfusion Defects,” Revista Portuguesa de Cardiologia 42 (2023): 519–524. 36893839 10.1016/j.repc.2022.07.018

[16] A. Jabbar , A. Pingitore , S. H. S. Pearce , A. Zaman , G. Iervasi , and S. Razvi , “Thyroid Hormones and Cardiovascular Disease,” Nature Reviews Cardiology 14 (2017): 39–55. 27811932 10.1038/nrcardio.2016.174

[17] A. L. Maia , I. M. Goemann , E. L. S. Meyer , and S. M. Wajner , “Type 1 Iodothyronine Deiodinase in Human Physiology and Disease,” Journal of Endocrinology 209 (2011): 283–297. 21415143 10.1530/JOE-10-0481

[18] G. Pasqualetti , V. Calsolaro , S. Bernardini , et al., “Degree of Peripheral Thyroxin Deiodination, Frailty, and Long‐Term Survival in Hospitalized Older Patients,” Journal of Clinical Endocrinology & Metabolism 103 (2018): 1867–1876. 29546287 10.1210/jc.2017-02149

[19] S. Wang , Y. Wang , S. Sun , et al., “Free Triiodothyronine to Free Thyroxine Ratio as a Marker of Poor Prognosis in Euthyroid Patients With Acute Coronary Syndrome and Diabetes After Percutaneous Coronary Intervention,” Frontiers in Endocrinology 15 (2024): 1322969. 38654927 10.3389/fendo.2024.1322969 PMC11036861

[20] C. Han , L. Wang , C. Liu , et al., “FT3/FT4 Enhances Risk Assessment in Patients With Non‐ST‐Segment Elevation Acute Coronary Syndrome Undergoing Percutaneous Coronary Intervention Based on GRACE 2.0 Score,” Angiology 76 (2025): 125–140. 37876209 10.1177/00033197231199228

[21] C. Wang , S. Han , Y. Li , F. Tong , Z. Li , and Z. Sun , “Value of FT3/FT4 Ratio in Prognosis of Patients With Heart Failure: A Propensity‐Matched Study,” Frontiers in Cardiovascular Medicine 9 (2022): 859608. 35498022 10.3389/fcvm.2022.859608 PMC9039517

[22] A. R. Leite , J. S. Neves , A. Angélico‐Gonçalves , et al., “Clinical and Pathophysiologic Insights of Free Triiodothyronine/Free Thyroxine Ratio in Patients With Heart Failure With Preserved Ejection Fraction: Data From the NETDiamond Cohort,” Cardiology 148 (2023): 239–245. 37285810 10.1159/000530136

[23] H. Zhang , W. Che , K. Shi , et al., “FT4/FT3 Ratio: A Novel Biomarker Predicts Coronary Microvascular Dysfunction (CMD) in Euthyroid INOCA Patients,” Frontiers in Endocrinology 13 (2022): 1021326. 36187090 10.3389/fendo.2022.1021326 PMC9520241

[24] S. Razvi , A. Jabbar , A. Pingitore , et al., “Thyroid Hormones and Cardiovascular Function and Diseases,” Journal of the American College of Cardiology 71 (2018): 1781–1796. 29673469 10.1016/j.jacc.2018.02.045

[25] A. Mancini , C. Di Segni , S. Raimondo , et al., “Thyroid Hormones, Oxidative Stress, and Inflammation,” Mediators of Inflammation 2016 (2016): 6757154. 27051079 10.1155/2016/6757154 PMC4802023

[26] M. Khan , S. Mukherjee , S. Bank , and S. Maiti , “Role of Thyroid Hormone and Oxidant Stress in Cardiovascular Diseases,” Endocrine, Metabolic & Immune Disorders‐Drug Targets 21 (2021): 1282–1288. 10.2174/1871530320666200917114501 32940193

[27] S. Debmalya , R. Saumitra , and M. H. Singh , “Interplay Between Cardiovascular and Thyroid Dysfunctions: A Review of Clinical Implications and Management Strategies,” Endocrine Regulations 56 (2022): 311–328. 36270343 10.2478/enr-2022-0033

[28] M. H. Sørensen , A. S. Bojer , J. R. N. Pontoppidan , et al., “Reduced Myocardial Perfusion Reserve in Type 2 Diabetes Is Caused by Increased Perfusion at Rest and Decreased Maximal Perfusion During Stress,” Diabetes Care 43 (2020): 1285–1292. 32193248 10.2337/dc19-2172

